# Cases Report the Cronkhite-Canada Syndrome: Improving the Prognosis

**DOI:** 10.1097/MD.0000000000002356

**Published:** 2015-12-31

**Authors:** Yi Qun Yu, Peter James Whorwell, Lin Heng Wang, Jun Xiang Li, Qing Chang, Jie Meng

**Affiliations:** From the Department of Gastroenterology, Dongfang Hospital, Beijing University of Chinese Medicine, Beijing, China (YQY, LHW, JXL, QC, JM); and Neurogastroenterology Unit, University Hospital of South Manchester, Manchester, UK (PJW).

## Abstract

Cronkhite-Canada syndrome (CCS) is a rare nongenetic polyposis syndrome first reported by Cronkhite and Canada in 1955.^1^ Up to the present time, the literature consists of ∼400 cases of CCS with the majority being reported from Japan^2^ although 49 cases have been described in China.^3^

CCS is characterized by diffuse polyposis of the digestive tract in association with ectodermal changes, such as onychomadesis, alopecia, and cutaneous hyperpigmentation. The principal symptoms of CCS are diarrhea, weight loss, abdominal pain, and other gastrointestinal complications, such as protein-losing enteropathy and malnutrition.

It has been traditional to consider that CCS is associated with a poor prognosis. This paper describes a relatively mild case and reviews the literature, which more recently, suggests that it may be a more benign condition that might actually be reversible with treatment.

There is some evidence that infection or disturbed immunity may be involved in the pathophysiology and that targeting such abnormalities could have therapeutic potential.

A strong case could be made for establishing an international case registry for this disease so that the pathophysiology, treatment, and prognosis could become much better understood.

## Patient Information

A previously healthy, 53-year-old, nonsmoking, nonalcohol drinking, female taxi driver presented to our department in July 2014 with an 8-month history of abdominal pain and weight loss.

In December 2013, she developed intermittent colicky pain in the left lower abdomen and started to note blood and purulent mucus in her stools. As her stools were positive for blood and white cells, a diagnosis of infective enteritis was made and she was treated with levofloxacin but this did not lead to any improvement.

In Feburary 2014, she was admitted to a local hospital for upper and lower gastrointestinal endoscopy. Gastroscopy revealed multiple protuberant lesions in antrum and erosions in duodenum. Colonoscopy showed multiple ulcers and polyps in the colon with biopsies being reported as sessile adenoma. She was diagnosed as having ulcerative colitis and treated with intravenous omeprazole 40 mg daily, mesalamine 3 g daily by mouth, and dexamethasone 10 mg of retention enemas daily. After 10 days, the abdominal pain and the mucopurulent bloody stools started to improve, but during this period she began to develop hyperpigmentation of both the hands and the feet. She also complained of fatigue and dysphagia and a second colonoscopy showed that the colitis and proctitis was persisting.

In March 2014, she was admitted to a local tertiary care general hospital. She was started on enteral nutrition but her abdominal pain and diarrhea got worse and she started to develop alopecia and her finger nails started to peel. Gastroscopy showed esophagitis, chronic superficial gastritis, multiple ulcers in the duodenum and a biopsy revealed chronic inflammation, stromal edema, and vascular proliferation.

In May 2014, the patient went to a tertiary gastroenterology hospital in order to try and make a definite diagnosis. The possibility of CCS was raised and she was also seen in the dermatology department where fungal infection was excluded and the nail changes were diagnosed as onycholysis. She was prescribed esomeprazole 20 mg p.o. Bid and Arsanyl, a kind of mucosal protective 100 mg p.o. Tid and went back to her hometown.

Between May and July, she also took Chinese herbal medicines and Arsanyl in addition to the proton pump inhibitor (PPI) but continued to suffer from epigastric discomfort and anorexia. The pigmentation faded gradually and the alopecia and onycholysisalso improved.

A repeat gastroscopy in July 2014 showed esophagitis, chronic superficial gastritis, and multiple gastric polyps.

### Clinical Findings

**FIGURE 1:**
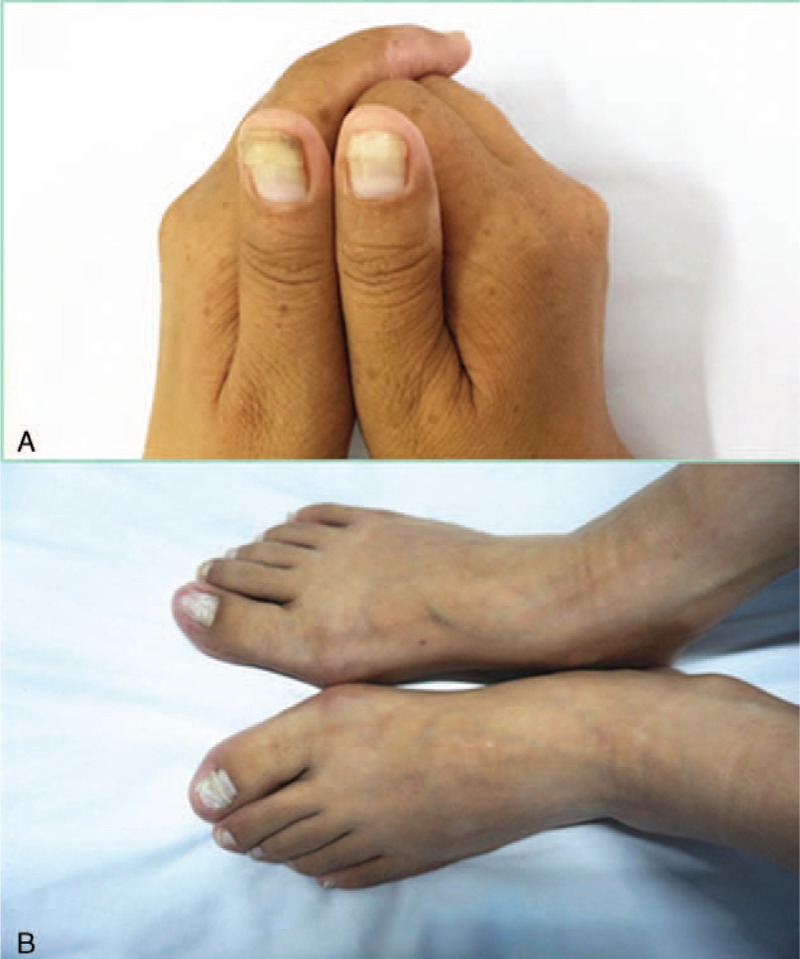
(A) Hyperpigmentation and onycholysis of the hands. (B) Hyperpigmentation and onycholysis of the feet.

She was admitted to our department in the late of July 2014 having lost 15 kg in 8 months. Physical examination revealed some tenderness of the left lower abdomen, slight alopecia, hyperpigmentation of the hands, especially over the metacarpophalangeal joints and onycholysis of both the hands and the feet (Fig. 1A and B).

### Diagnostic Assessment

Stool and urine routine tests were normal with a negative fecal immunochemical test of occult blood. Hematology and all immunoglobulins were normal. A screen for autoantibodies, including antinuclear antibody, was negative and liver and renal function tests were normal. Serum albumin was 38.5 g/L, low-density lipoprotein LDL was 1.84 (2.07–3.37) mmol/L, and serum iron was 47.8 (7.3–23.6) μmol/L. Carcinoembryonic antigen (CEA), NSE, CA19–9, CA125, CA724, CA153 were all normal although Cyfra211 was slightly elevated 4.48(0.1–3.3) ng/mL. TSH was slightly elevated 4.31(0.27–4.2) μIU/mL, but T3, T4, FT3 and FT4 were all normal.

ECG, chest x-ray were normal and abdominal ultrasound revealed a fatty liver. Ultrasound of the thyroid showed diffuse thyroid nodules. Carbon-13 breath test (Rapid Urease Test) for *Helicobacter pylori* was negative.

**FIGURE 2:**
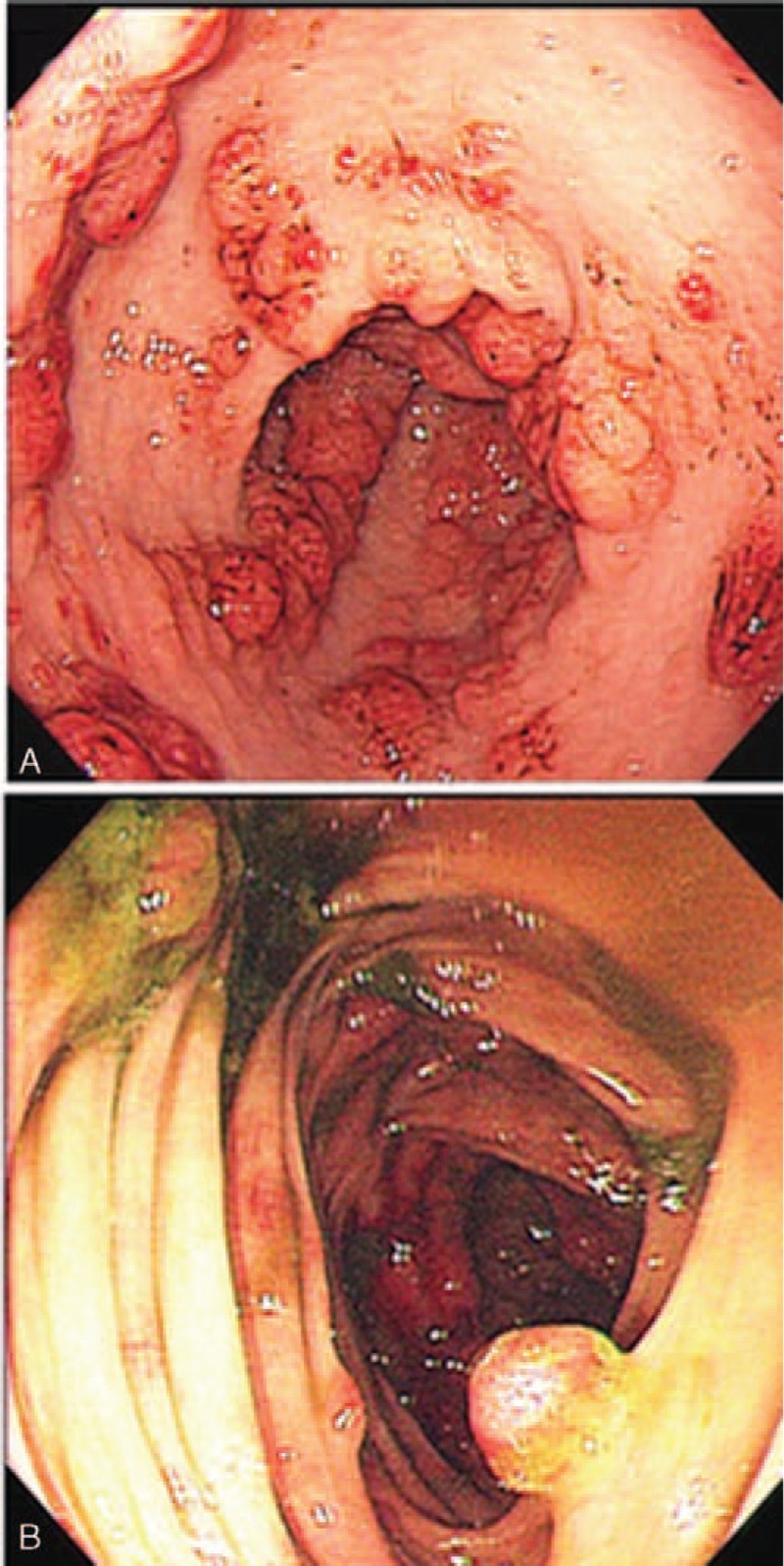
(A) Endoscopic appearance of the stomach showing numerous polypoid lesions with nodular edematous mucosa throughout the stomach. (B) Endoscopic appearance of the colon showing multiple polyps.

**FIGURE 3:**
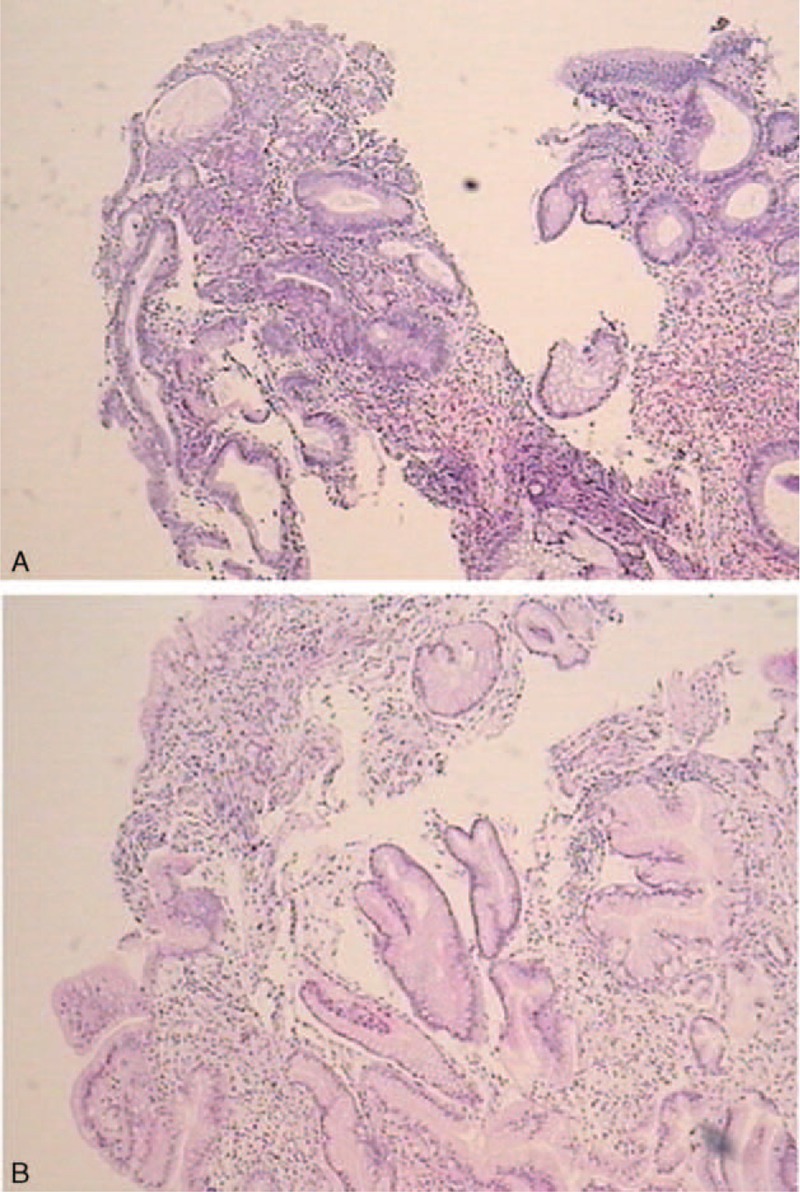
(A) Biopsies from polyps in the antrum showing chronic inflammation in mucosa, stromal hyperemia, and edema, and focal hyperplasia. (B) Biopsies from polyps in the colon showing a mixture of inflammatory cells consisting of lymphocytes and neutrophil granulocytes and clusters of epithelioid cells, crypt abscesses, stromal edema and hyperemia, infiltration of eosinophile granulocytes, and focal hyperplasia of epithelial cells.

Upper GI radiography: irregular mucosa of the antrum and duodenal bulb with nodular filling defects. Gastroscopy: numerous polypoid lesions with nodular edematous mucosa throughout the stomach and duodenum (Fig. 2A), no polyps found in the esophagus. Biopsies from the antrum: chronic inflammation in mucosa, stromal hyperemia and edema, and focal hyperplasia (Fig. 3A). Colonoscopy: multiple polyps were found in the cecum, ascending colon, sigmoid colon, and rectum as well as erosions (Fig. 2B). No polyps were found in the terminal ileum. Biopsies showed a mixture of inflammatory cells consisting of lymphocytes and neutrophil granulocytes and clusters of epithelioid cells, crypt abscesses, stromal edema and hyperemia, infiltration of eosinophile granulocytes, and focal hyperplasia of epithelial cells (Fig. 3B).

### Therapeutic Intervention

The patient was then diagnosed as CCS according to her symptoms, endoscopic findings, and pathology report. We prescribed Chinese herbal medicines and Arsanyl 100 mg tid in addition to esomeprazole 20 mg p.o. Bid for her. The treatment principle of Chinese herbal medicines was nourishing the liver and kidney according to the theory of traditional Chinese medicine, the chief medicinal was prepared rehmannia root, Radix Rehmanniae Praeparata.

## DISCUSSION

### Clinical Manifestations

Cronkhite-Canada syndrome (CCS) is characterized by polyposis, which can occur anywhere in the gastrointestinal tract except the esophagus. Initially CCS was considered to be a hamartomatous polyposis syndrome, but it is now thought that the polyps can additionally be inflammatory, adenomatous, and hyperplastic in type. Other clinical manifestations of CCS include ectodermal changes, such as onychomadesis, alopecia, and cutaneous hyperpigmentation. Symptoms include diarrhea, weight loss, abdominal pain, taste abnormalities, and features of malnutrition. Anemia, serum electrolyte disturbances, and hypoproteinemia are common in CCS patients as a result of chronic diarrhea and malnutrition. Osteoporotic fractures may result from malabsorption of calcium or prolonged glucocorticoid therapy or both ^3^ and death can occur for a variety of reasons including severe infection and malignant change. Goto divided the disease into 5 types according to the principal symptom:^2^ Diarrhea (type 1), dysgeusia (type 2), sensory abnormalities in the mouth accompanied by thirst (type 3), abdominal symptoms other than diarrhea (type 4), and alopecia as a predominant symptom (type 5). All patients must have gastrointestinal polyposis and hyperpigmentation although there is a report of a possible case of CCS without polypsis but severe atrophy and diffuse marked edema of the gastric and duodenal mucosa.^4^ As ectodemal change and vitiligo are prominent, scalp biopsy has shown noninflammatory loss of follicular units, miniaturization of the hair shafts, markedly dilated follicles, and heavy deposition of glycosaminoglycans in the reticular dermis.^5^

The initial symptoms of our case were abdominal pain, purulent diarrhea with rectal bleeding. Consequently, she would have been classified as type I, as the other features including the partial loss of taste and the ectodermal changes, such as onychomadesis, alopecia, and hyperpigmentation, appeared later. It was interesting to note that in our case, following treatment with mesalamine and dexamethasone, the abdominal pain and diarrhea were the first features to be relieved. The improvement in onychomadesis and regrowth of hair followed later raising the possibility that the first features to appear maybe the first to improve with treatment.

### Etiology

The cause of CCS is completely unknown and there is no evidence that it is a hereditary disease other than 1 case report of the condition occurring in a 50-year-old man and his son.^6^ There have been reports of increased levels of IgG4 or IgG4 containing plasma cells in the polyps of CCS ^7,8^ although this could not be confirmed in another study where out of 7 cases only 1 showed an increased IgG4-positive plasma cells. Consequently, the current evidence for some form of immune abnormality is not sufficient to draw any firm conclusions.

Some cases have been reported to be associated with hypothyroidism.^9^ In our case the TSH was minimally elevated 4.31 μIU/mL (0.27–4.2) although the T3, T4, Free T3, and Free T4 were all normal. Interestingly thyroid nodules were found on ultrasound examination.

There is also some preliminary work from Asia suggesting there may be some relationship with *H. pylori* infection.^10–12^ However, in view of the ubiquitous distribution of this organism in Asia it would be of interest to assess the prevalence of CCS in geographical areas with a low prevalence of *H. pylori.*

### Treatment

There is no specific treatment for CCS although a variety of approaches have been tried on an empirical basis.

Symptomatic and supportive treatment is important and may lead to improvement.^13^ This includes nutritional support with high-protein supplements or enteral feeding, correction of anemia and electrolyte imbalance, replacement of any mineral or vitamin deficiencies, as well as treatment of any associated infection.^14–16^ Proton pump inhibitors may also be worth prescribing.^11^

Progressive remission, including regression of the polyps, after *H. pylori* eradication has been reported ^10–12^ although in some of the reports it is not clear what the *H. pylori* status of the patients was before the start of treatment. CCS does appear to be common in Asia where the prevalence of *H. pylori* is high and it is noteworthy that there is a report incriminating this organism as a risk factor for the development colorectal adenomas.^17^ However, our patient had a negative carbon-13 breath hydrogen test and therefore was not given *H. pylori* eradication therapy.

In view of reports of a possible immunological basis for CCS, immunosuppression by corticosteroids or long-term azathioprine has been reported to lead to improvement.^7,8,18^ Corticosteroids are more frequently used to maintain remission although they carry the risk of osteoporotic fracture.^3^ Not surprisingly, there have been no controlled trials assessing the optimal dosage or duration of treatment with corticosteroids or azathioprine for the treatment of CCS. Furthermore, following any remission, it is still uncertain whether medication should be continued and if so, at what dose. The possibility of using mesalazine has also been explored with the suggestion that some patients might improve with this drug or sulphasalazine .^15,19^ In cases where there is a failure to respond to steroids, cyclosporine may be worth considering.^20^ In addition, a recent study has shown that TNF may play an important role in the development of CCS. Consequently, infliximab may be worth considering and Watanabe and colleagues have tried this drug in a patient suffering from frequent relapses with high levels of tumor necrosis factor alpha (TNF-α) in tissue affected by CCS. They showed that after 20 months of treatment the patient experienced a complete remission with disappearance of the polyposis.^21^ The role of surgery in the treatment of CCS is unclear, but it may have to be considered if complications develop.^16^ Adenomas found at endoscopy should be removed and followed up by surveillance endoscopy to facilitate early detection of any malignancies which can then be treated appropriately.

## PROGNOSIS

The literature suggests that CCS has a poor prognosis with Poulson and colleagues suggesting a 5-year mortality of 55%.^22^ Furthermore there also seems to be a relatively high risk of malignancy at a variety of sites both inside and outside the gastrointestinal system. However, because the condition is so rare, estimates of survival rates and the prevalence of cancer in CCS are unlikely to be very accurate. However, as there seems to be some evidence that better treatment regimes are being developed for CCS these figures might be expected to improve.

There seems little doubt that the majority of cancers in CCS are in the gastrointestinal with the suggestion that ∼14 % of patients develop this complication.^23^ Gastric cancer seems to be the most common malignancy with a Japanese study reporting a prevalence of 10.2%. Some cases had multiple gastric cancers of the stomach which tended to be well differentiated and often limited to the submucosa. ^24,25^ Carcinomas also occur in colon ^26^ and rectum.^27,28^ Serrated adenomas appear to account for 40% of the polypoid lesions in CCS which is much higher than their prevalence in polyps in the general population and patients with colorectal cancer associated with CCS frequently have polyps containing serrated adenomas.^29^

Interestingly, patients with CCS seem to have a tendency to develop extra-intestinal cancer, such as cholangiocellular carcinoma,^30^ giant cell bone tumor, ^31^ and lung cancer.^32^ In addition, some cases of CCS have been associated with cancer in >1 organ with one report describing cancer in the esophagus, stomach, and lung ^32^ and another concomitant esophageal and gastric cancers.^33^

Steroids and anti-TNF treatment seems to show promise in the treatment of CCS and there is a report of raised CEA levels falling after treatment with steroids. In addition, if the disappearance of polyps previously reported following the use of an anti-TNF ^21^ can be confirmed, this suggests that these drugs should perhaps be introduced at an earlier stage. Obviously, there is a strong need for long-term follow-up studies in order to determine whether some of the more encouraging treatment results reported recently are maintained over time. In 1 case of CCS, the CEA level increased remarkably and then decreased following prednisolone treatment. Whether steroid or anti-TNF therapy can lower the risk of carcinomas in the long term will need longer follow-up of patients taking this type of medication.

In view of the fact that malignant change in the gastrointestinal system is a significant risk in CCS, appropriate screening needs to be put in place, although there are currently no guidelines on how this should be done and at what frequency.

## CONCLUSION

Cronkhite-Canada syndrome is a rare nonhereditary polyposis condition, which needs to be differentiated from other polyposis syndromes such as Peutz–Jeghers syndrome, familial adenomatous polyposis, juvenile polyposis syndrome, and Cowden syndrome. This should not be difficult when all the other features are present but when they appear later, as in our case, the diagnosis can be more difficult to establish.

The observation that remission can sometimes be induced by the eradication of *H. pylori* suggests that some form of infection might be important in the pathogenesis of CCS. However, not all cases are associated with *H. pylori* infection, raising the possibility that the antibiotic combination used for the eradication of *H. pylori* might be clearing another, not yet identified, organism. Perhaps a lesson needs to be learned from Whipples disease and the subsequent identification *Tropheryma whipplei*.

Another possible mechanism that has been implicated in the pathogenesis of CCS is autoimmunity. Some support for this hypothesis comes from reports that the condition seems to improve with immunosuppressive therapy such as corticosteroids, azathioprine, cyclosporine, and more recently anti-TNF treatment. However, it remains to be determined whether anti-infectives or immunosuppressives should be used alone and, if so, which is best. Furthermore, some form of a combination of these 2 approaches might achieve even better results than the 2 treatments alone.

It has been traditional to consider that CCS is associated with a poor prognosis and a 5-year mortality of 55% after diagnosis has been reported.^22^ However, in the cases reported from China, the mortality has not been so high and, so far, our patient's illness has been relatively mild. In addition, with the advent of treatment modalities that seem to have a significant impact on the course of the disease, it is likely that the outlook will improve further.

Rather than relying on a series of case reports with variable follow-up, a strong case could be made for the introduction an international CCS patient registry to enable the prognosis and treatment of CCS to be far more accurately established.

### Informed Consent

The patient has given written informed consent for her case to be reported. Because it was not a clinical trial and no off-label drugs was used, the ethical approval is not necessary for this case report.
